# Shikimic acid (SA) inhibits neuro-inflammation and exerts neuroprotective effects in an LPS-induced *in vitro* and *in vivo* model

**DOI:** 10.3389/fphar.2023.1265571

**Published:** 2023-11-02

**Authors:** Xueying Bao, Zhuangzhuang Zheng, Jincai Lv, Jindian Bao, Sitong Chang, Xin Jiang, Ying Xin

**Affiliations:** ^1^ Department of Radiation Oncology, The First Hospital of Jilin University, Changchun, China; ^2^ Jilin Provincial Key Laboratory of Radiation Oncology and Therapy, The First Hospital of Jilin University, Changchun, China; ^3^ NHC Key Laboratory of Radiobiology, School of Public Health, Jilin University, Changchun, China; ^4^ Key Laboratory of Pathobiology, Ministry of Education, and College of Basic Medical Science, Jilin University, Changchun, China

**Keywords:** shikimic acid, parkinson’s disease, microglia, neuro-inflammation, neuroprotection

## Abstract

Numerous studies have shown that neuroinflammation is involved in the process of neuronal damage in neurodegenerative diseases such as Parkinson’s disease (PD), for example, and that inhibiting neuroinflammation help improve PD. Shikimic acid (SA) has anti-inflammatory, analgesic and antioxidant activities in numerous diseases. However, its effect and mechanism in PD remain unclear. In this experiment, we found that SA inhibits production of pro-inflammatory mediators and ROS in LPS-induced BV2 cells. Mechanistic studies demonstrated that SA suppresses neuro-inflammation by activating the AKT/Nrf2 pathway and inhibiting the NF-κB pathway. Further *in vivo* study, we confirmed that SA ameliorated the neurological damage and behavioral deficits caused by LPS injection in mice. In summary, these study highlighted the beneficial role of SA as a novel therapy with potential PD drug by targeting neuro-inflammation.

## Introduction

Parkinson’s disease, the world’s second most neurodegenerative disease, has a high incidence of 1%–2% among people over 60 years of age. The causes of Parkinson’s disease are complex and it is widely believed that genetic, environmental and physiological aging are important causes of PD (Samii et al., 2004; Reich and Savitt, 2019). During PD, denaturation of nigra dopaminergic neurons and decreased striatal dopamine levels. It then showed that the motor activity decreased, and in severe cases, resting tremor, myotonia and other motor dysfunction. Some also have non-motor symptoms such as memory loss and depression. Neuroimmune and inflammatory responses are involved in the course of PD. Neuro-inflammation-mediated neurotoxicity is extremely important in the cascade of neuronal death in PD (Jankovic and Tan, 2020; Sarkar et al., 2020). Neuro-inflammation is characterised by the presence of large numbers of activated microglia around damaged neurons. During the disease process, microglia maintain chronic inflammation through self-propelled circulatory pathways that release inflammatory mediators and ROS. However, persistent inflammation can lead to the accumulation of inflammatory mediators and ROS, damaging peripheral neurons and exacerbating the process of PD (Hirsch and Hunot, 2009; Pajares et al., 2020; Williams et al., 2021). Therefore, suppressing neuro-inflammation can somewhat reduce neuronal damage and mitigate the progression of PD.

Microglia are the main effector cells of neuroinflammation (Feldman, 2022; Pottorf et al., 2022). Pathological neuroinflammation associated with neurodegeneration is primarily mediated by microglia, which are resident immune cells of the CNS (Han et al., 2021; Leng and Edison, 2021). Aggregation of misfolded proteins is a hallmark of many neurodegenerative diseases and leads to cytotoxicity. Most protein misfolding errors result in functionally acquired or dominant inactivation effects. Microglia sense and internalize misfolded proteins to facilitate clearance, but in neurodegenerative disease states, this process is dysregulated, leading to neuroinflammation (Calsolaro and Edison, 2016; Teleanu et al., 2022). During PD, microglia accumulate around the injured neurons and activation occurs. Early microglia activation mediating acute inflammation may play beneficial roles, such as clearing damaged tissue as well as toxic substances. However, continued activation of microglia mediates chronic inflammation, leading to the accumulation of pro-inflammatory mediators that in turn exacerbate neuronal damage (Ho, 2019; Badanjak et al., 2021). Thus inhibition of microglia-mediated chronic inflammation may be important for the treatment of inflammation-related neurological disorders.

Shikimic acid (SA) is a monomeric compound extracted from the Chinese herb star anise, naturally occurring in the dried, ripe fruit of the magnolia plant star anise, *etc.* Anise, produced in China and Vietnam, is a small oriental tree of the magnolia family that bears fruits with a pungent, liquorice-like flavour and is a traditional condiment in oriental cooking and the main spice in liqueurs such as French green aniseed wine and aniseed liqueur. It is also generally used to add flavour to sweets, aniseed and tobacco (Patra et al., 2020; Shi et al., 2021; Rodriguez Cerro et al., 2022). SA, a monomeric compound derived from anise, has been reported to have a wide range of pharmacological effects. Studies have shown that SA has anti-inflammatory, analgesic and antioxidant activities and that it improves colitis in rats by inhibiting the NF-κB pathway, inhibiting the production of pro-inflammatory mediators and reducing oxidative stress (Xing J. F. et al., 2012; Xing J. et al., 2012; Sun et al., 2016). In mice, SA inhibited LPS-induced cellular pro-inflammatory cytokines and attenuated mechanical nociceptive sensitization in mice (Rabelo et al., 2016). In addition, SA exerts a protective effect on osteoarthritic cartilage by restoring impaired autophagy and inhibiting the MAPK/NF-κB signalling pathway (You et al., 2021). The above studies reveal the role of SA in peripheral tissue inflammation. It has also been shown that SA promotes the differentiation of oligodendrocyte precursor cells and accelerates myelin regeneration in mice (Lu et al., 2019), suggesting its therapeutic potential in central nervous system disorders. However, its effects on neuro-inflammation have not been reported. We therefore aimed to investigate the effect of SA on neuro-inflammation and its potential role in PD.

## Materials and methods

### Reagents

Shikimic acid (SA) was obtained from Shanghai yuan ye Biotech Co., Ltd (Shanghai, China). The purity is ≥98%. Dimethyl sulfoxide (DMSO) and Lipopoly-saccharide (LPS) were obtained from Sigma Aldrich (St Louis, MO, United States). Serum (FBS), Trypsin (0.05%), Penicillin-Streptomycin Solution (PS) and Dulbecco’s modified eagle’s medium (DMEM) were purchased from Gibco (Grand Island, NY, United States). RA (nrf2 inhibitor), JSH-23 (NF-κB inhibitor) and MK2206 (AKT inhibitor) were purchased from Selleck Chemicals Aldrich (Shanghai, China). In our experiments we used MK2206、RA or JSH treatments to inhibit the activation of the AKT、Nrf2 and NF-κB pathways in cells, with pretreatment concentrations and times of MK2206 (10 μM) for 4 h、RA (5 μM) for 4 h and JSH (1 μM) for 3 h. The concentration and duration of pretreatment were referred to previous studies (Peng et al., 2019; Gao et al., 2020; He et al., 2021a).

### Cell experiment

A mouse microglia (BV2 cells, from Jin Yuan Biotechnology Co. (Shanghai, China)) was cultured with DMEM medium containing 10% FBS and 1% PS (the condition: air atmosphere, 95%; carbon dioxide (CO_2_), 5% and temperature, 37°C). When density reaches 90%, cells were processed and passaged with 0.05% trypsin.

### CCK-8 assay

BV2 cells growing in good condition were inoculated into 96-well plates at a density of 2–3 × 10^5^. After 12 h, different concentrations of SA (2.5 μM、5 μM、10 μM、20 μM、40 μM) were added into well and cultured for another 20 h. After that, the medium was replaced with fresh DMEM without FBS and cultured for another 2 h. Subsequently CCK8 (Shang Bao Biological, Shanghai, China) was added and incubated for another 3 h. Finally, absorbance was measured with a microplate reader (BioTek, Winooski, VT, United States) at 450 nm.

### NO detection assay

NO level in the culture media was assayed with the Griess reagent (Beijing Solarbio Science and Technology Co., Beijing, China) according to the manufacturer’s instructions. Briefly, BV-2 cells were inoculated in the 12-well plates at a density of 2–3 × 10^4^. After 12 h, cells were treated with SA and/or LPS for 24 h. The supernatant (100 μL) was mixed with an equal volume of Griess reagent (parts 1 and 2) and incubated in the dark for 30 min. After that, the absorbance were measured with a microplate reader (BioTek, Winooski, VT, United States) at 530 nm and NO concentrations were calculated based on the standard curve of sodium nitrite.

### ROS detection assay

Release of ROS in BV2 cells is detected using ROS detection kits (Thermo Fisher Scientific Inc., Shanghai, China). Briefly, BV-2 cells were inoculated in the 96-well plates at a density of 2–3×10^5^. After 12 h, cells were treated with SA and/or LPS for 24 h. The medium was replaced with DMEM without FBS and DCFH-DA (10 μM) was added to each well, and then incubated for 30 min at 37°C. After that, the absorbance were measured with a microplate reader (BioTek, Winooski, VT, United States) under the condition: excitation wavelength 488 nm and emission wavelength 525 nm.

### Western blot assay

Expression of associated proteins in tissues and cells is detected using the Western blot method. Firstly, proteins are cleaved using NP40 reagents (Beyotime Biotechnology, Shanghai, China) and concentrations are quantified using BCA kits (Beyotime Biotechnology, Shanghai, China). Subsequently, Electrophoresis (120 V, 1.5 h) was performed with 50 μg of protein. After electrophoresis, proteins were transferred to PVDF membranes (Millipore, Billerica, MA, United States) (70 V, 2 h). After closed for 3 h in 5% skim milk, the membrane was incubated with the primary antibody (iNOS (1:1,000), p-NF-κB p65 (1:1,000), p-IκB (1:1,000),β-actin (1:10,000), TH (1:1,000), p-AKT (1:2000), Nrf2 (1:5,000), iba-1 (1:500), PCNA (1:5,000), COX2 (1:1,000), IκB (1:1,000), NF-κB p65 (1:2000), β-actin (1:10,000)) at room temperature for 4 h and the secondary antibody (1:4,000) for 2 h at room temperature. The primary antibodies are from Proteintech (Wuhan, China) and Cell Signaling Technology (Massachusetts, United States). The secondary antibody is from Invitrogen (California, America). After that, the membranes were developed using ECL ultrasensitive luminescent solution (Yeasen Biotechnology Co., Shanghai, China) and imaged using Kodak X-ray film (Ruike Medical Equipment Co., Xiamen, China). Results are analyzed using ImageJ software.

### Quantitative PCR assay

The mRNA expression of inflammatory mediators in tissues and cells is detected using quantitative PCR methods. Briefly, RNA was extracted using trizol reagent (Sigma Aldrich, Shanghai, China). Then, 500 ng of RNA was reverse transcribed into cDNA using a reverse transcription kit (Yeasen Biotechnology, Shanghai, China). Next, cDNA is amplified using the 2×M5 Hi per PCR Super mix (Yeasen Biotechnology, Shanghai, China) and Cq values are recorded with Bio Rad system. Finally, the mRNA level of the mediators was assessed relative to β-actin according to the 2^−ΔΔCT^. Primer sequences of the mediators refer to previous studies (Hou et al., 2018).

### ELISA

BV2 cells growing in good condition were seeded into 24-well plates at a density of 1–2×10^4^. After 12 h, the culture medium was replaced and SA and/or LPS were added. After continuing incubation for 24 h, the culture medium was collected and the expression of IL-6 and TNF-α was tested using ELISA kits (Bio Legend, San Diego, CA, United States) according to the manufacturer’s protocol.

### Animal experiment

Eight weeks-old mice (C57/BL6) were purchased from Chang sheng Biological Company (Changchun, China). Mice were allowed 2 weeks to adapt after being purchased. The mice were housed in a normal temperature environment (temperature of 22°C ± 2°C and humidity of 40%–60%) with free intake of food and water. The animal experimental process is presented in Figure 5A. Briefly, mice were randomly divided into four groups: Saline group (Injection of saline in the SN), SA group (Intraperitoneal drug delivery SA) single-injection LPS group (Injection of LPS in the SN) and SA + LPS (Injection of LPS in the SN and Intraperitoneal drug delivery SA). LPS (3 μL) dissolved in saline (concentration for 3 mg/mL) was injected into the SN ((anteroposterior (AP) = 5.2 mm, lateral (LAT) = 2.1 mm and dorsoventral (DV) = 7.8 mm)). The injection rate is 0.3 μL/min. SA (100 mg/kg) dissolved in saline is administered by intraperitoneal injection as once a day from LPS injection. Concentrations of SA used are referenced to previous studies (Rabelo et al., 2016; Lu et al., 2019). Four weeks later, behavioral tests and other experiments are performed. All animal experiments were approved by the Ethics Committee of the First Hospital of Jilin University and performed in strict accordance with the relevant provisions of the Guidelines for the Care and Use of Animals in Animal Laboratories (Declaration of Helsinki 2013).

### Behavioral evaluation

After 4 weeks of administration, the mental state and motor activity of mice were evaluated by mental state scoring, rod climbing, rod turning and open field test. Behavioral evaluation methods refer to previous articles (He et al., 2021b; Snyder et al., 2021). The brief methodology is as follows:

Mental state scoring: After modeling, the mice were scored by observing their mental state. Scoring criteria: 0 points for no adverse performance; 1 point for mild reduction of autonomous activity and slightly slow reaction; On top of the symptoms of 1 point, a significant decrease in voluntary activity, loss of balance, unsteady gait, and bowed back are recorded as 2 points; On top of the symptoms of 2 points, 3 points were recorded for symptoms such as weakness of both hind limbs, splitting of hind limbs after lifting the tail, and involuntary trembling of the body; On top of the symptoms of 3 points, 4 points were recorded for symptoms such as paralysis of both hind limbs, inability to stand, increased muscle tone in the extremities, and cessation of eating; Rod climbing: Mice were placed at the top of a wooden pole about 60 cm long and 1–2 cm in diameter so that they could move freely. The timing started when the hind limbs of the mice left the top of the pole and stopped when the head touched the bottom. The time taken for the mice to climb from the top to the bottom was recorded. Each mouse was repeated 3 times and the average was calculated.

Rod turning: Mice were placed on a rotor bar fatigue apparatus. The speed of the rotor bar was adjusted to approximately 40 revolutions per minute. The time that the mice remained on the rotor bar fatigue apparatus without falling was recorded. Each mouse was repeated 3 times and the average was calculated.

Open field test: Mice were placed in a 50 cm × 50 cm × 30 cm open field box and allowed to move freely. A camera was used to record the trajectory of the mice running in the open field area within 5 min. The total distance run by the mice in 5 min and the time of entry into the central region were analyzed using Top Scan software (Any-maze, Stoelting Co.).

### Immunohistochemical (IHC) staining

The mice’s dopaminergic neurons and microglia in the SN were detected using IHC staining. These experimental procedures refer to previous study. Briefly, after being euthanized, the midbrain tissue of the mice was acquired and fixed. The IHC process is then performed using Ultrasensitive TMS-P kit (Beyotime Biotechnology, Shanghai, China) according to the manufacturer’s protocols. The dopaminergic neurons were labeled with the anti-tyrosine hydroxylase (TH) antibody (1:200, Abcam, Cambridge, United Kingdom) and microglia were labeled with the anti-ionized calcium binding adapter molecule 1(IBA-1) antibody (1:200, Abcam, Cambridge, United Kingdom). The numbers of TH and IBA-1 positive cells were counted and averaged separately by three investigators unrelated to this experiment were recorded.

### Data analyses

All data are presented as mean ± SEM and analyzed using Graph Pad Prism 9. The normality of data distribution was verified by Shapiro-Wilk test. Homocedasticity of variance was verified by Brown-Forsythe test (for multiple groups). Differences between treatment groups were compared using a combination of one-way ANOVA and Tukey’s multiple comparisons test. *p* < 0.05 were considered statistically significant.

## Results

### SA inhibits production of NO and ROS in LPS-stimulated BV2 cells

**FIGURE 1:**
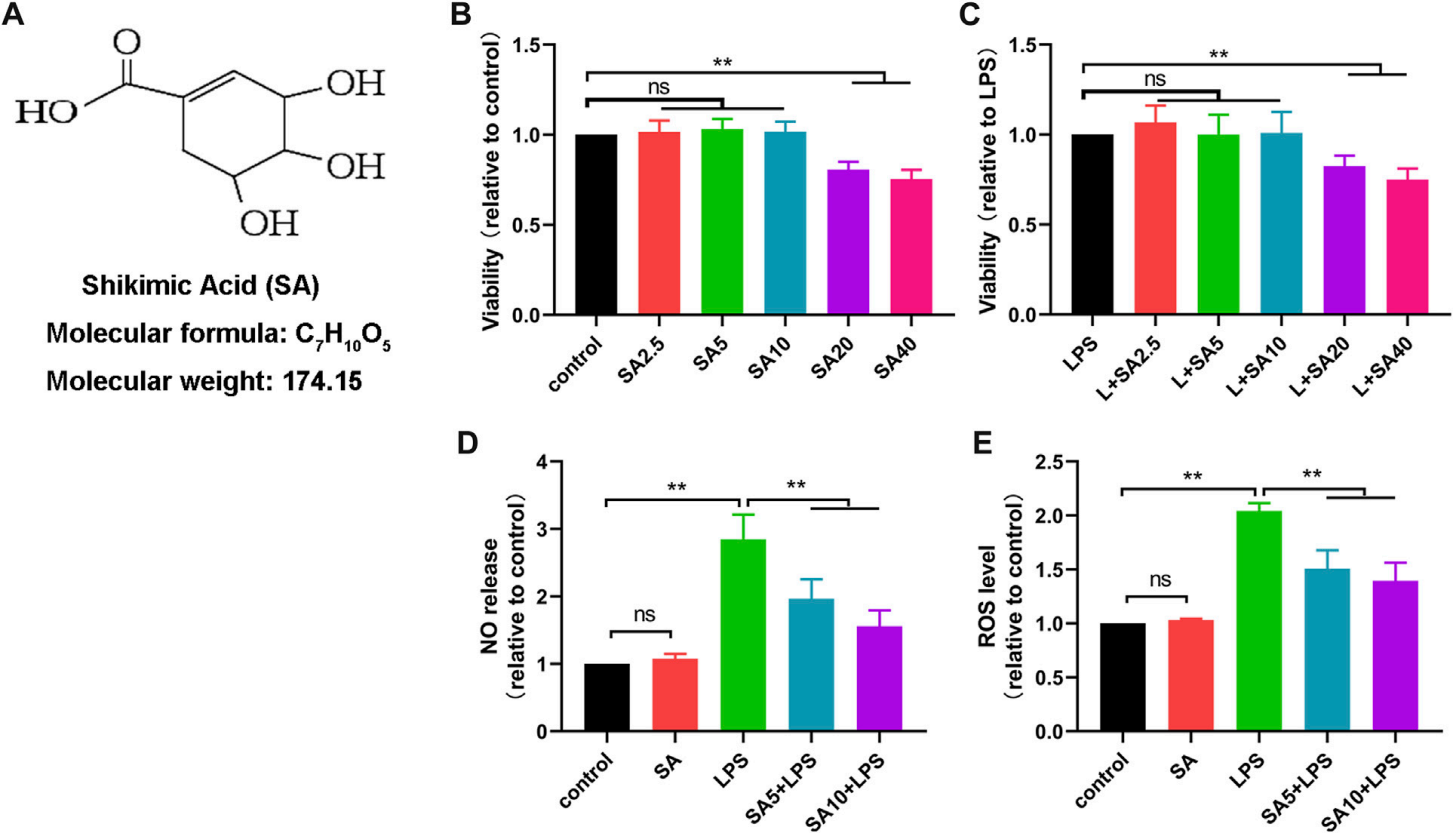
SA inhibits production of NO and ROS in LPS-stimulated BV2 cells. **(A)** Molecular weight and chemical structure formula of SA. B, **(C)** The effect of SA (2.5 μM、5 μM、10 μM、20 μM、40 μM) on the survival of BV2 cells was examined by the CCK8 method. **(D)** The effect of SA on NO release in BV2 cells was assayed by the NO assay. **(E)** The effect of SA on ROS release in BV2 cells was assayed by the ROS assay. Results are shown as mean ± SEM (n = 4). **p < 0.01 stands for biologically significant difference. *NS* stands for No Significant Difference.

To determine the effect of SA on neuroinflammation, we cultured microglial cell lines (BV2) *in vitro*. Firstly, we investigated the changes of BV2 cell survival rate after different concentrations of SA treatment (2.5 μM、5 μM、10 μM、20 μM、40 μM) via CCK8 assay. Results showed that SA not exceeding 10 μM did not have a significant effect on the viability of BV2 (Figures 1B, C ). Therefore, we chose 5 μM and 10 μM SA for the follow-up study.

Next, we treated BV2 with SA (5 μM and 10 μM) and LPS (1 μg/mL) for 24 h, and investigated the effect of SA on NO release and ROS production in LPS-exposed BV2 cells via NO and ROS detection assay. Results showed that SA inhibited the release of NO (Figure 1D) and the production of ROS (Figure 1E) in BV2 cells.

### SA activates AKT, Nrf2 pathways in BV2 cells

**FIGURE 2:**
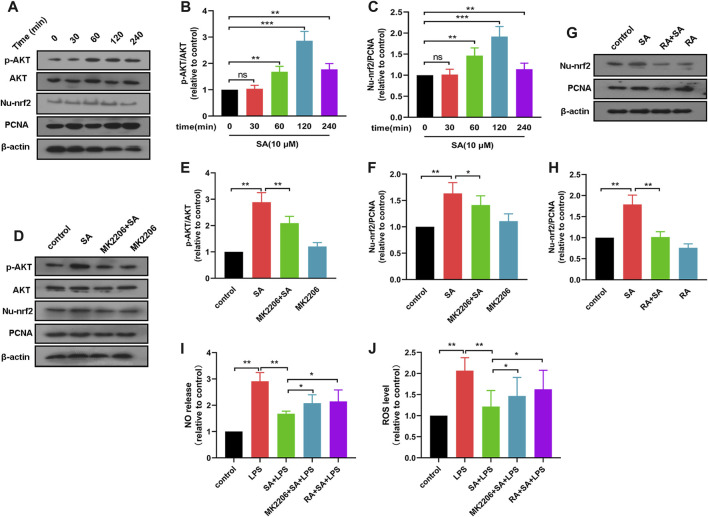
SA inhibits production of NO and ROS through activation of the AKT/Nrf2 pathway. **(A–C)** After SA treatment for different times (0 min, 30 min, 60 min, 120 min, 240 min), the activation of AKT and Nrf2 in BV2 were examined by Western blot method. **(D–F)** The effect of SA on AKT and Nrf2 pathway activation after MK2206 treatment was detected by Western blot method. **(G, H)** The effect of SA on Nrf2 pathway activation after RA treatment was examined by Western blot method. **(I, J)** The effect of SA on NO and ROS release after MK2206 and RA treatment was assayed by NO and ROS detection methods. Results are shown as mean ± SEM (n = 4). *p < 0.05, **p < 0.01 and ***p < 0.001 stands for biologically significant difference. *NS* stands for No Significant Difference.

It was shown that NO release and ROS production were regulated by AKT and Nrf2 pathways. To further elucidate the role of SA, we investigated the effect of SA on AKT and Nrf2 pathways in BV2 cells. Results displayed that SA promoted the activation of AKT and Nrf2 pathways in BV2 (Figures 2A–C).

Subsequently, we treated BV2 with AKT inhibitor MK2206 (10 μM) for 4 h and then examined the effect of SA on the activation of AKT pathway and Nrf2 pathway in BV2 cells. Results displayed that MK2206 inhibited the activation of Nrf2 pathway by SA (Figures 2D–F). These results demonstrate that SA promotes the activation of the Nrf2 pathway through the AKT pathway. After that, we treated BV2 with Nrf2 inhibitor RA (5 μM) for 4 h, and then examined the activation of Nrf2 by SA. Results showed that RA inhibited the activation of Nrf2 by SA (Figures 2G, H).

Finally, we also investigated the effect of SA on NO release and ROS production in BV2 cells after pretreatment of BV2 cells with MK2206 or RA. Results displayed that MK2206 or RA pretreatment reversed the effect of SA on NO and ROS in BV2 cells to some extent (Figures 2I, J). These results demonstrate that SA inhibits NO release and ROS production through activation of the AKT/Nrf2 pathway.

### SA inhibits the expression of pro-inflammatory mediators in BV2 cells

**FIGURE 3:**
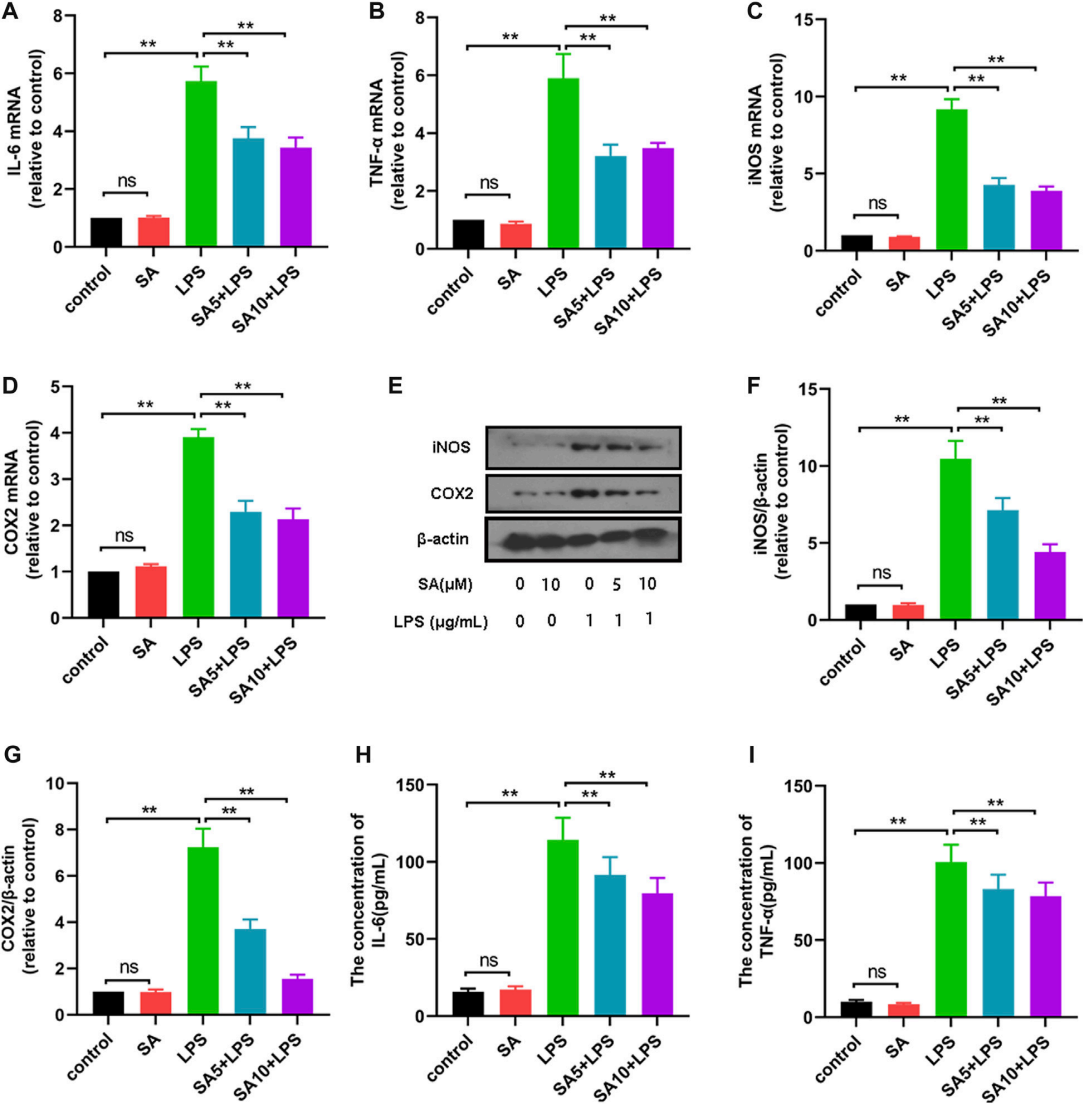
SA inhibits the production of pro-inflammatory mediators in LPS-stimulated BV2 cells. **(A–D)** The mRNA expression of pro-inflammatory mediators (IL-6, TNF-α, iNOS and COX2) was examined by quantification PCR assay. **(E–G)** The protein expression of pro-inflammatory mediators (iNOS and COX2) was examined by Western blot assay. **(H, I, G)**: The protein expression of pro-inflammatory mediators (IL-6, TNF-α) was examined by ELISA assay. Results are shown as mean ± SEM (n = 4). *p < 0.05, **p < 0.01 stands for biologically significant difference. *NS* stands for No Significant Difference.

To further elucidate the role of SA, we investigated the effect of SA on the production of inflammatory mediators in BV2 cells. First, BV2 cells were treated with SA (5 μM and 10 μM) and LPS (1 μg/mL) for 12 h, and the mRNA expression of inflammatory mediators were detected using a quantitative PCR method. Results displayed that SA inhibited mRNA expression of inflammatory mediators (IL-6 (Figure 3A), TNF-α (Figure 3B), iNOS (Figure 3C) and COX-2 (Figure 3D)) in LPS-stimulated BV2 cells. Subsequently, BV2 cells were treated with SA (5 μM and 10 μM) and LPS (1 μg/mL) for 24 h, and the protein expression of inflammatory mediators were detected using ELISA and Western blot methods. Results displayed that SA inhibited protein expression of inflammatory mediators (iNOS (Figures 3E, F), COX-2 (Figures 3E, G), IL-6 (Figure 3H) and TNF-α (Figure 3I)). These results demonstrate that SA inhibits the production of pro-inflammatory mediators in LPS-stimulated BV2 cells.

### SA inhibits the activation of the NF- κB pathway in LPS-stimulated BV2 cells

**FIGURE 4:**
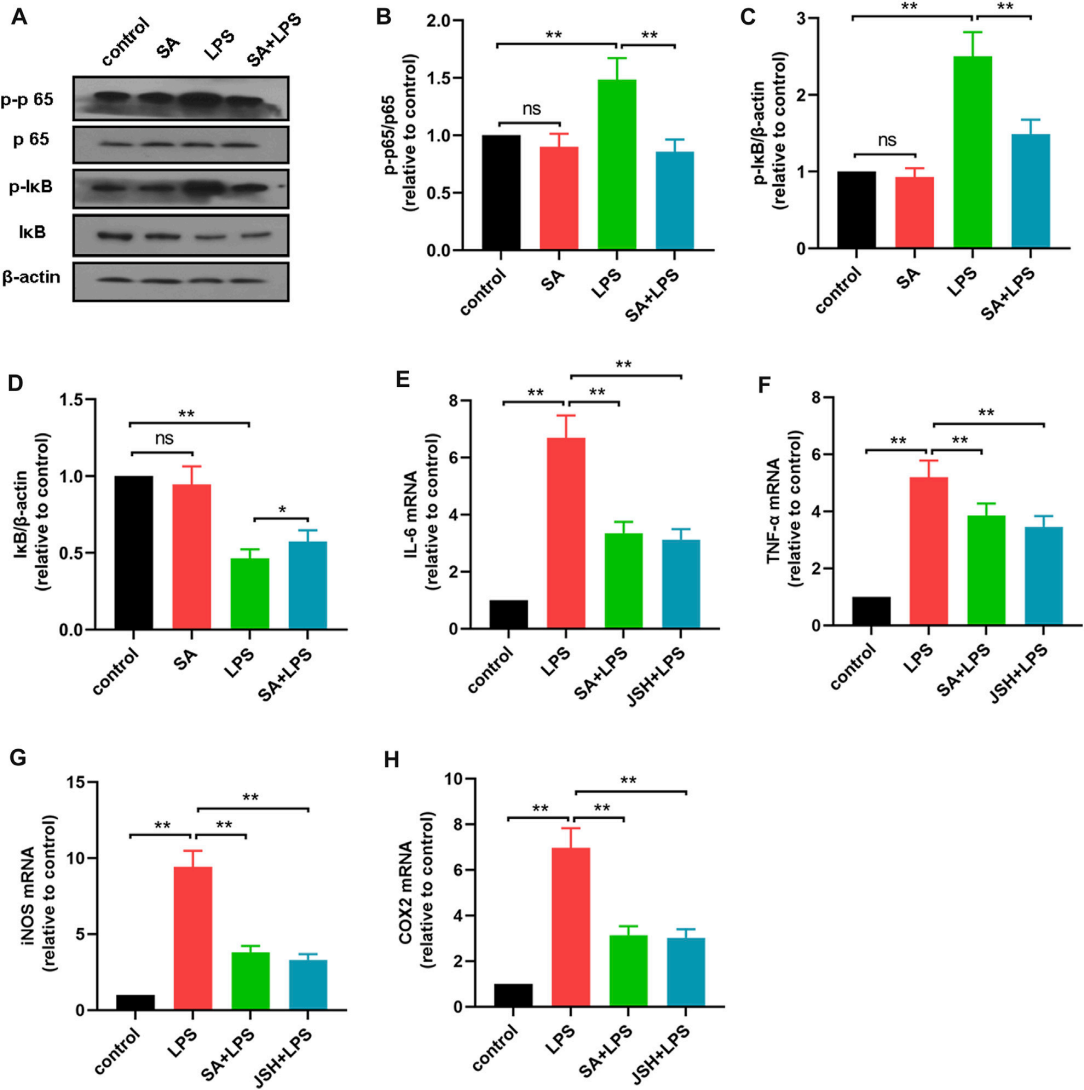
SA inhibits the activation of the NF-κB pathway in LPS-stimulated BV2 cells. **(A–D)** The effect of SA on NF-κB pathway in LPS-exposed BV2 cells was examined by Western blot method. **(E–H)** The effect of SA and JSH on the expression of pro-inflammatory mediators (IL-6, TNF-α, iNOS and COX2) in BV2 cells was detected by quantitative PCR method. Results are shown as mean ± SEM (n = 4). **p < 0.01 stands for biologically significant difference. *NS* stands for No Significant Difference.

NF-κB pathway is a key pathway of inflammation, and to elucidate potential mechanism by which SA regulates inflammatory mediators, we further examined the effect of SA on NF-κB activation. First, BV2 cells were treated with SA (10 μM) and LPS (1 μg/mL) for 1 h, and the changes in p65, IκB and its phosphorylated proteins were detected using Western blot method. Results showed that SA inhibited the phosphorylation of IκB (Figures 4A, B), p65 (Figures 4A, C) and the degradation of IκB (Figures 4A, D).

Subsequently, BV2 cells were treated with the NF-κB inhibitor JSH (100 μM) and then the effect of SA on inflammatory mediator production were investigated. Results showed that SA inhibited the production of inflammatory mediators in BV2 cells in the same way as JSH (Figures 4E–H). These results demonstrate that SA inhibits the production of inflammatory mediators in BV2 cells in the same way as JSH.

### SA administration ameliorates clinical status and dyskinesia in mice exposed to LPS

**FIGURE 5:**
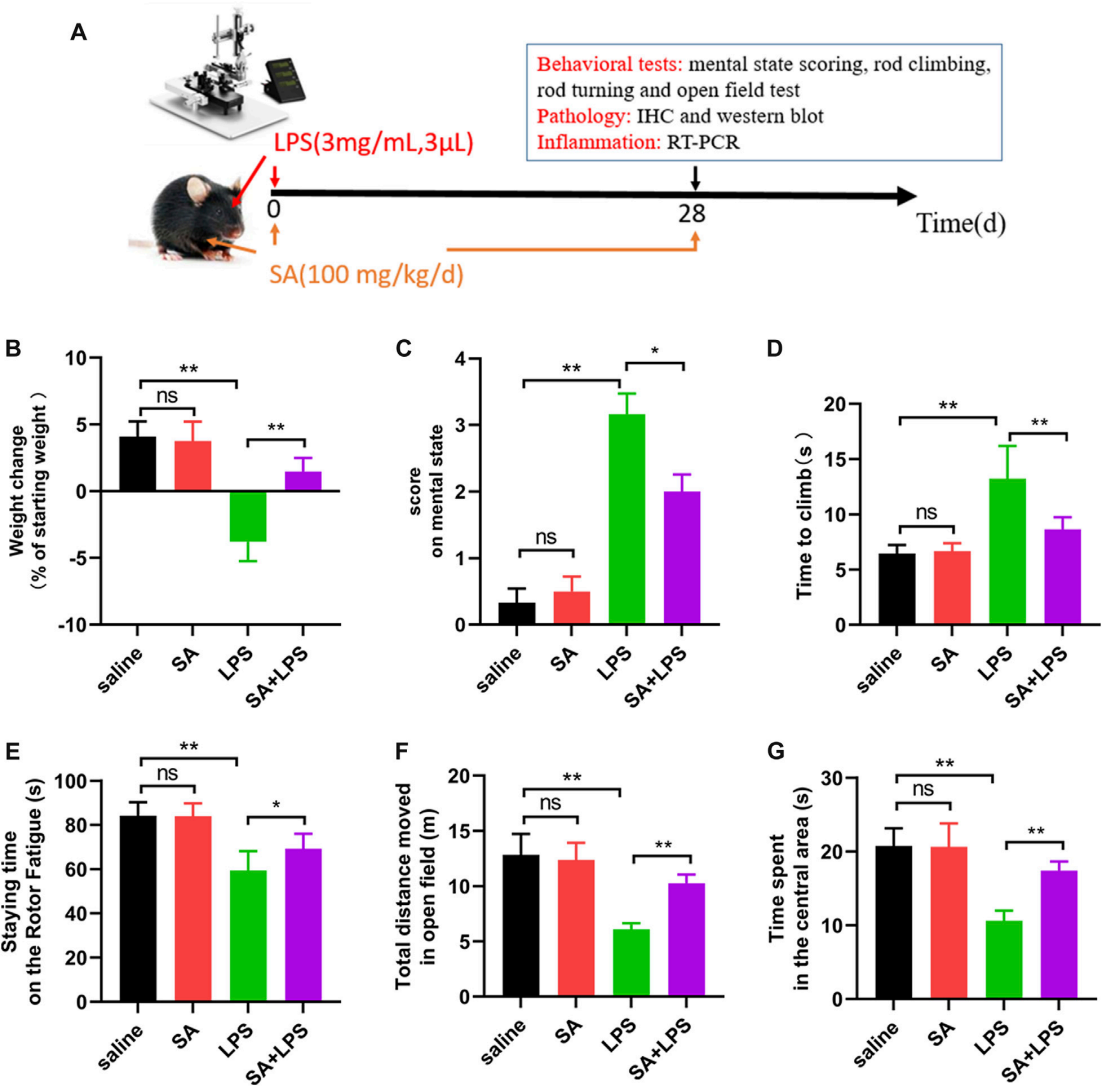
SA administration ameliorates clinical status and dyskinesia in mice exposed to LPS. **(A)** Flow chart of animal experiment. **(B)** Changes in body weight of mice before and after the experiment. **(C)** Mental status score of mice. **(D)** The time required to climb from the top to the bottom in the mouse pole climbing experiment. **(E)** The time that mice persist on the spinning bar fatigue meter without dropping. **(F, G)** Total distance moved and time spent exploring the central area in open-field experiments in mice. Results are shown as mean ± SEM (n = 6). **p < 0.01 and *p < 0.05 stands for biologically significant difference. *NS* stands for No Significant Difference.

In clinic, PD mainly manifests in the loss of motor ability, some with depressed, depression and other mental symptoms. In order to further study the effect of SA, we injected LPS to SN of mice and administered SA, and studied the effect of SA on clinical state and motor behavior in mice with LPS injection (Figure 5A). First, we tested the weight changes in mice before and after the experiment and showed that the SA treatment ameliorated the weight loss caused by the LPS injection (Figure 5B). Subsequently, we focused on the mice’s mental status, motor level and muscle tremor to score the mice for clinic pathology and found that SA treatment reduced the clinic pathology scores of LPS-injected mice (Figure 5C).

Next, to further elucidate the effect of SA on the locomotor behaviour of mice, we investigated the effect of SA on the locomotor ability of LPS injected mice through behavioural experiments. The results of the pole climbing experiment displayed that SA treatment ameliorated the reduction in balance in mice caused by LPS injection (Figure 5D). The results of the rotating bar experiment displayed that SA treatment ameliorated the reduction in fatigue tolerance in mice caused by LPS injection (Figure 5E). The results of the open-field experiments displayed that SA treatment improved the reduction in locomotion and the ability to explore unknown areas in mice caused by LPS injection (Figures 5F, G).

In summary, the above results illustrate that SA administration ameliorates clinical status and dyskinesia in mice exposed to LPS.

### SA administration ameliorates dopaminergic neuron loss in mice exposed to LPS

**FIGURE 6:**
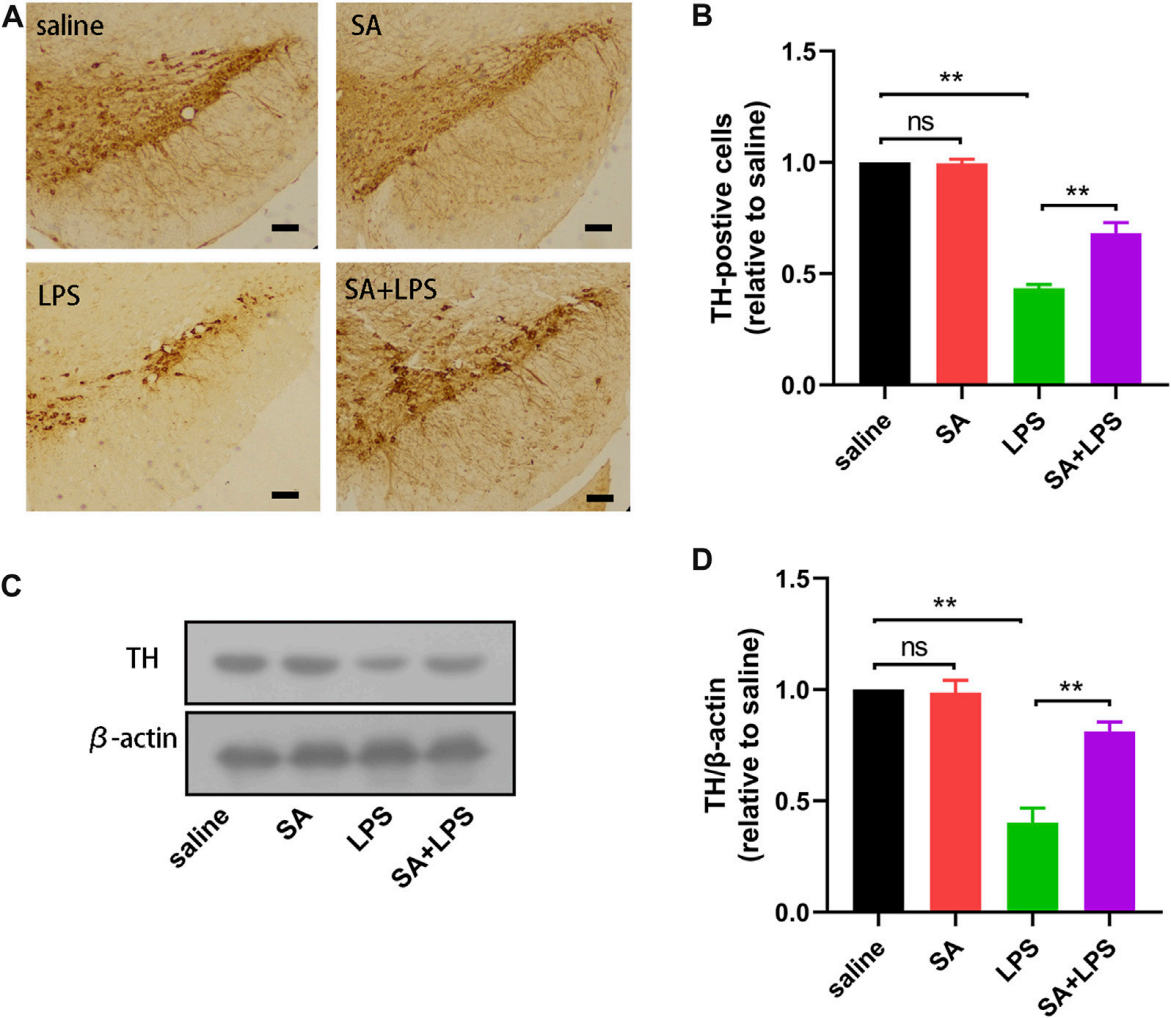
SA administration ameliorates dopaminergic neuron loss in mice exposed to LPS. **(A, B)** The number of TH-positive cells in the SN of mice is measured by immunohistochemical staining methods (The bar stands for 50 μm). **(C, D)** Expression of TH protein in the mice middle brain is measured by the Western blot method. Results are shown as mean ± SEM (n = 4). **p < 0.01 stands for biologically significant difference. *NS* stands for No Significant Difference.

The main pathological change in PD is the degenerative absence of dopaminergic (DA) neurons in the SN. To further elucidate the neuroprotective effects of SA *in vivo*, we investigated the effects of SA on DA neurons in the SN of LPS-injected mice. The results of immunohistochemistry showed that SA treatment alleviated the reduction of TH-positive cells in the SN of mice caused by LPS injection (Figures 6A, B). We then examined the changes of TH protein in the mice midbrain by Western blot. Results displayed that SA restrained the reduction of TH protein in the mice midbrain caused by LPS injection (Figures 6C, D). These results prove that SA administration ameliorates dopaminergic neuron loss in mice exposed to LPS.

### SA administration inhibits microglia over-activation in mice exposed to LPS

**FIGURE 7:**
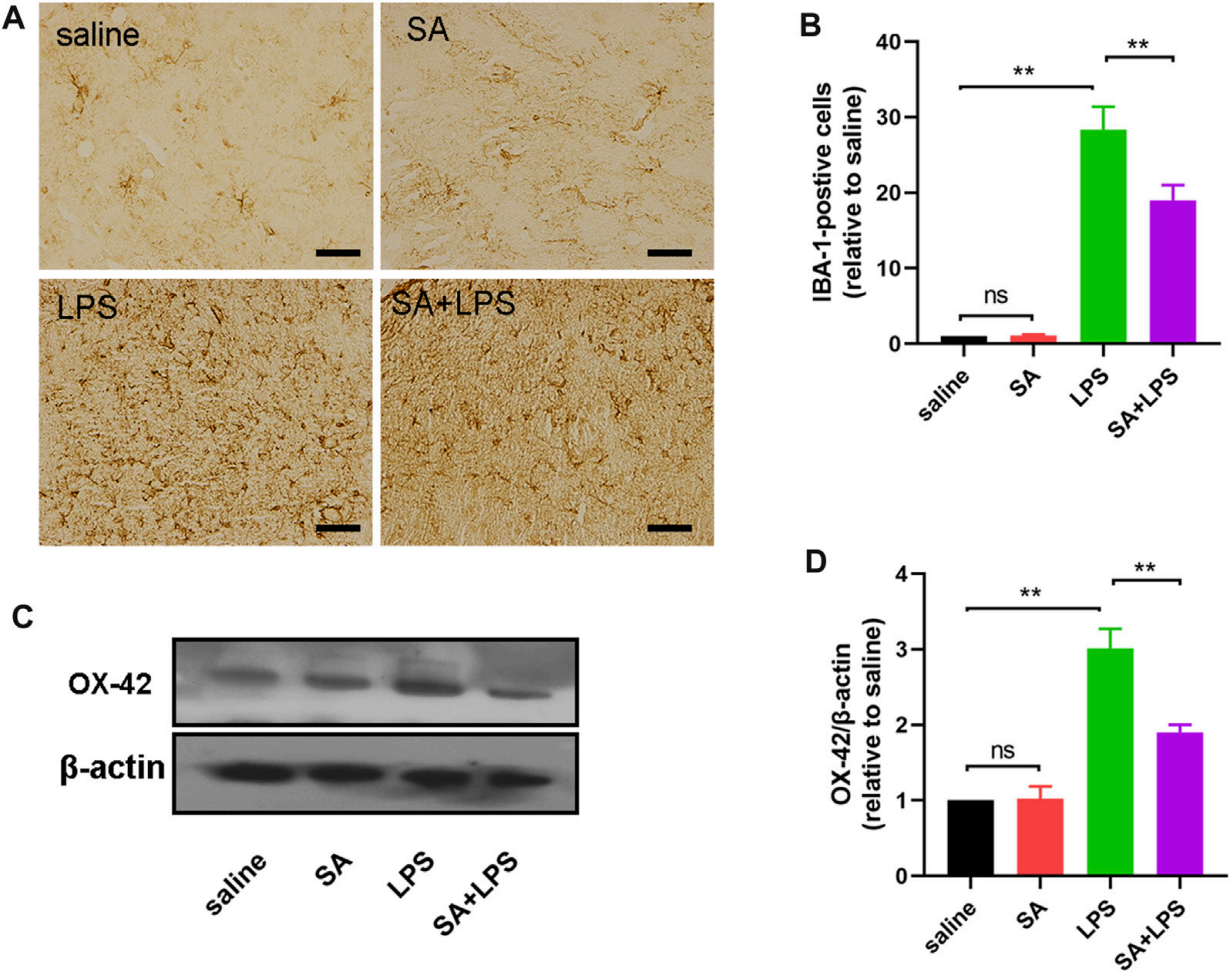
SA administration inhibits microglia over-activation in mice exposed to LPS. **(A, B)** The number of IBA-1-positive cells in the SN of mice is measured by immunohistochemical staining methods (The bar stands for 20 μm). **(C, D)** Expression of OX-42 protein in the middle brain of mice is measured by the Western blot method. Results are shown as mean ± SEM (n = 4). **p < 0.01 stands for biologically significant difference. *NS* stands for No Significant Difference.

PD patients have large numbers of activated microglia in the SN of the midbrain, which are also the main effector cells of neuro-inflammation. To further elucidate the role of SA *in vivo*, we also investigated the effect of SA on microglia activation in the SN of LPS-injected mice. First, we examined the number of IBA-1-positive cells in the SN of the mouse midbrain using immunohistochemistry. The results showed that SA treatment reduced the number of IBA-1-positive cells in the SN of the brain in LPS-injected mice (Figures 7A, B). We then examined changes in OX-42 protein in the midbrain of mice by Western blot. The results showed that SA treatment reduced the expression of OX-42 protein in the midbrain of mice (Figures 7C, D). These results prove that SA administration inhibits microglia over-activation in mice exposed to LPS.

### SA administration inhibits inflammatory responses in the midbrain region in mice exposed to LPS

**FIGURE 8:**
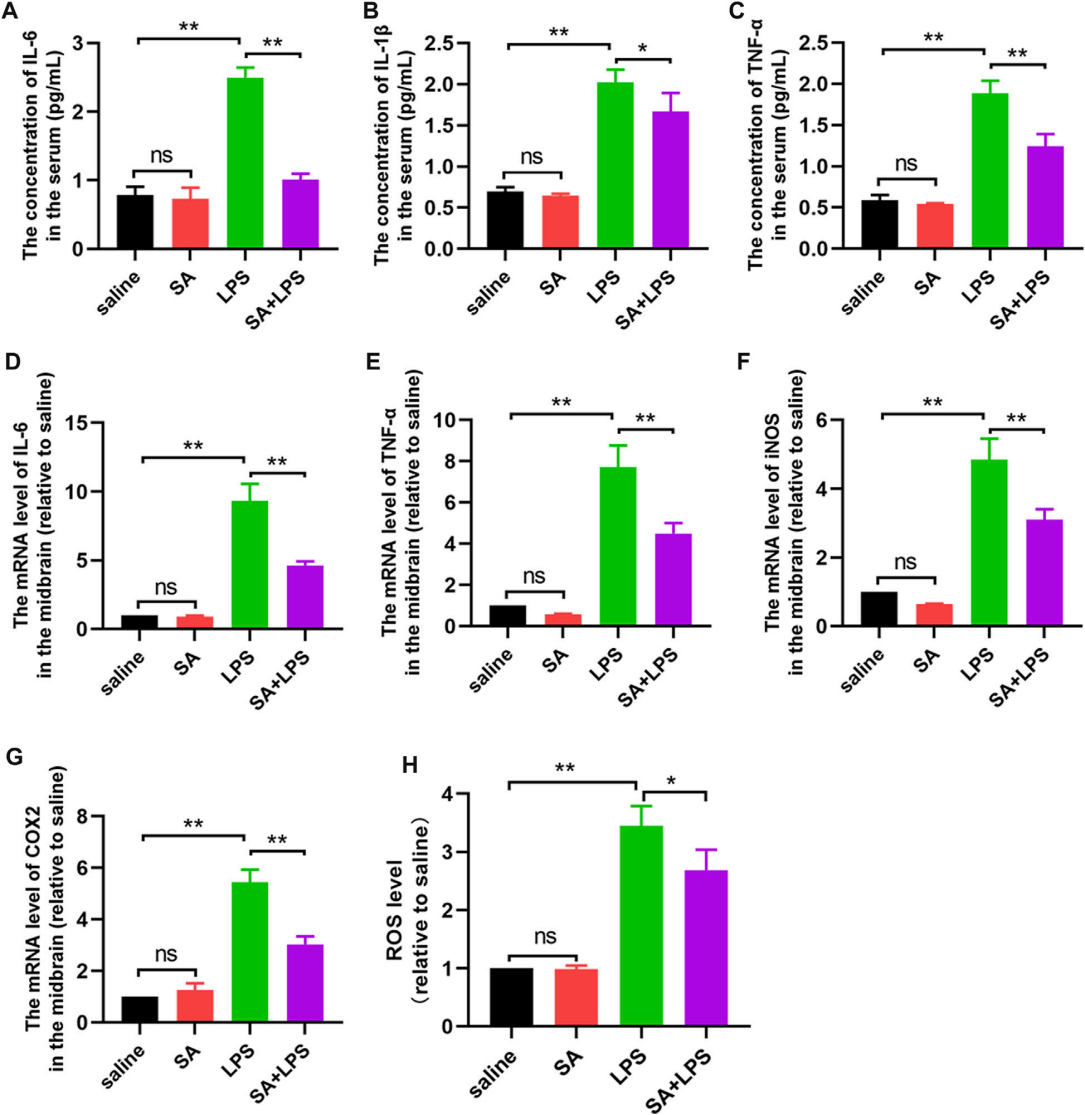
SA administration inhibits inflammatory responses in the midbrain region in mice exposed to LPS. **(A–C)** Protein expression of pro-inflammatory factors (IL-6, IL-1β and TNF-α) in the middle brain of mice is measured through the ELISA method. **(D–G)** The mRNA expression of pro-inflammatory mediators (IL-6, TNF-α, iNOS and COX2) in the middle brain of mice is measured through the qPCR method. **(H)** ROS expression in the midbrain of mice was measured through a ROS kit. Results are shown as mean ± SEM (n = 4). **p < 0.01 and *p < 0.05 stands for biologically significant difference. *NS* stands for No Significant Difference.

LPS is typically an inflammation-inducing factor and neuro-inflammation is related in the process of PD. Therefore, we next investigated the effect of SA on the inflammatory response in the brain of LPS-injected mice. Firstly, we measured the protein levels of pro-inflammatory factors (IL-6, IL-1β and TNF-α) in the mouse midbrain by ELISA. Results displayed that SA inhibited the protein expression of pro-inflammatory factors (Figures 8A–C) in the midbrain of LPS-injected mice. Afterwards, we examined the changes of pro-inflammatory mediator (IL-6, TNF-α, iNOS and COX2) mRNA in the midbrain of mice by qPCR. Results displayed that SA inhibited the mRNA expression of pro-inflammatory mediator (Figures 8D–G) in the midbrain of LPS-injected mice. Afterwards, we examined the effect of SA on ROS in the brain of LPS-injected mice. Results displayed that SA treatment inhibited the expression of ROS in the brain of LPS-injected mice (Figure 8H). These results prove that SA administration inhibits inflammatory responses in the midbrain region in mice exposed to LPS.

### SA administration activates the AKT pathway and inhibits the NF-κB pathway in the midbrain region in mice exposed to LPS

**FIGURE 9:**
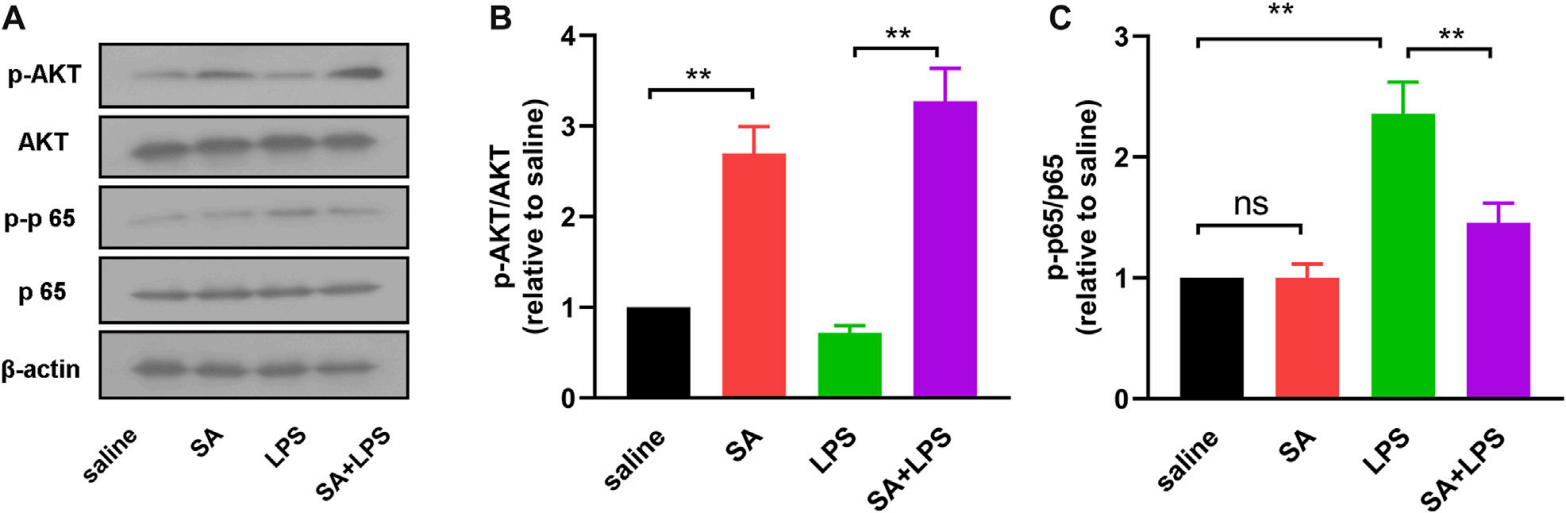
SA administration activates the AKT pathway and inhibits the NF-κB pathway. **(A, B)** AKT and phosphorylated AKT in mouse midbrain were detected by Western blot method. **(A, C)** NF-κB p65 and phosphorylated NF-κB p65 in mouse midbrain were detected by Western blot method. Results are shown as mean ± SEM (n = 4). **p < 0.01 stands for biologically significant difference. *NS* stands for No Significant Difference.

To further clarify the mechanism of SA’s inhibitory neuroinflammatory effects. We next examined the activation of AKT and NF-κB pathways in the midbrain of mice by Western blot. The results showed that the phosphorylation level of AKT in the midbrain of mice in the SA + LPS group was increased and the phosphorylation level of NF-κB p65 was decreased compared with that in the LPS-injected group (Figure 9). These results demonstrate that SA administration activates the AKT pathway and inhibits the NF-κB pathway in the midbrain region in mice exposed to LPS.

## Discussion

**FIGURE 10:**
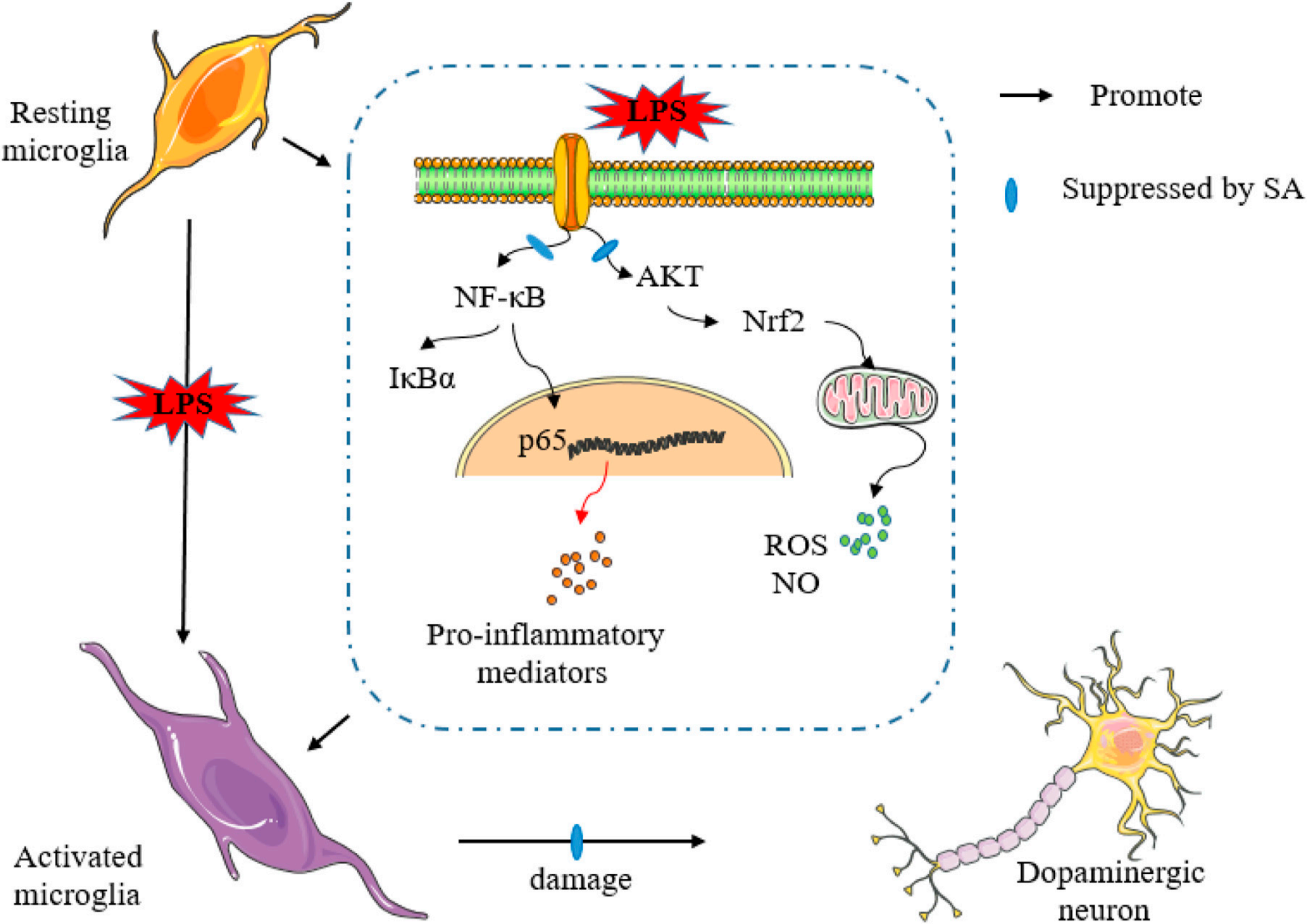
SA inhibits neuro-inflammation and exerts neuroprotective effects in an LPS-induced *in vitro* and *in vivo* model.

In this study, results confirmed the truth that SA inhibits neuro-inflammation and exerts neuroprotective effects in an LPS-induced *in vitro* and *in vivo* model (Figure 10). *In vitro* studies, SA suppressed the expression of inflammatory markers such as NO, ROS and pro-inflammatory mediators. Further mechanistic studies displayed that SA exerted anti-inflammatory effects in BV2 cells through activating AKT, Nrf2 pathway and inhibiting NF-κB pathway. *In vivo* studies, results showed that SA ameliorated the weight loss and motor impairment caused by LPS injection in mice. Furthermore studies displayed that SA reduced dopaminergic neuronal damage and inhibited microglia hyper-activation in the SN. In addition, results also showed that SA treatment suppressed the inflammatory response of midbrain in the LPS-injected mice.

Neuro-immune and inflammatory responses are related in the process of PD (De Virgilio et al., 2016; Heidari et al., 2022). Microglia are the main effector cells of neuro-inflammation (Pottorf et al., 2022). LPS is an inflammation-inducing factor (Catorce and Gevorkian, 2016). In this experiment, we investigated the effect of SA on LPS-induced inflammatory responses in microglia *in vitro*. First, we found that SA suppressed the production of NO and ROS in BV2 cells (Figure 1). AKT and Nrf2 are involved in the release of ROS (Liu et al., 2020; Liu et al., 2021), so we next examined the effect of SA on the AKT and Nrf2. Results displayed that SA treatment promoted the activation of AKT and Nrf2 pathways in BV2 cells (Figure 2). Further studies also found that SA inhibited the release of NO and ROS through activation of the AKT, Nrf2 pathway (Figure 2). These studies suggest a potential mechanism for the role of SA in microglia, namely, the inhibition of NO and ROS production via the AKT/Nrf2 pathway. It also suggests the possibility of SA alleviating inflammation-related diseases by targeting the AKT/Nrf2 pathway.

To further demonstrate the effect of SA on neuro-inflammation, we next studied the effect of SA on the release of pro-inflammatory mediators in microglia. Results displayed that SA treatment suppressed the expression of pro-inflammatory mediators in BV2 cells (Figure 3). Nuclear factor-κB (NF-κB) is a class of nuclear transcription factors present in the cytosol that, when induced by a variety of factors *in vivo* and externally, can be transferred to the nucleus and participate in the regulation of the expression of a variety of inflammatory genes (Liang et al., 2018; Zusso et al., 2019). To elucidate the mechanism by which SA suppressed pro-inflammatory mediators, we then examined the effect of SA on NF-κB pathway activation. The results showed that SA treatment suppressed the nucleation of p65 and the degradation and phosphorylation of IκBα (Figure 4). In addition, the results also showed that treatment with JSH, an inhibitor of NF-κB, also inhibited the production of pro-inflammatory mediators in BV2 cells, further demonstrating that SA regulates the expression of pro-inflammatory mediators in BV2 cells through the NF-κB pathway (Figure 4). These results suggest that SA may have the potential to inhibit neuro-inflammation and alleviate inflammation-related diseases by targeting the NF-κB pathway.

The main pathological feature of PD is the degenerative absence of DA neurons in the SN, which in turn leads to motor dysfunction. Patients with PD clinically present with some motor dysfunction such as decreased motor ability and balance (Emre et al., 2007; Bencs et al., 2020). In this experiment, we injected LPS into the midbrain SN of mice, and assessed changes in clinical signs after administration of SA. The results showed that SA treatment reduced the weight loss and elevated clinical mental status scores in mice caused by LPS injection (Figure 5). Subsequently, we further tested for behavioural changes in the mice. The results showed that SA administration improved balance and fatigue tolerance in the mice. In addition, the open field test also showed that SA administration improved locomotion and exploration in mice (Figure 5). These results suggest that SA treatment has an ameliorative effect on the loss of motor function in mice caused by LPS injection. Further to investigate why SA improves motor behaviour in mice, we next assessed the effects of SA on dopaminergic neuronal damage in the SN of the mouse midbrain. Results showed that SA treatment significantly inhibited the dopaminergic neuronal damage in the SN of the mouse midbrain caused by LPS injection (Figure 6).

LPS is an inflammation-inducing factor and neuro-inflammation is involved in the process of PD (Sampson et al., 2016; Grotemeyer et al., 2022). It is therefore reasonable to believe that the improvement of neuronal damage and motor deficits in LPS-injected mice by SA may be related to its anti-neuroinflammatory effects. To demonstrate this, we next investigated the effect of SA on microglia activation in the SN of LPS-injected mice. Results showed that SA treatment significantly suppressed the hyper-activation of microglia in the SN region of the brain in LPS-injected mice (Figure 7). We also examined changes in the inflammatory response in the midbrain of mice. Results showed that SA treatment suppressed the inflammatory response in the midbrain of LPS-injected mice (Figure 8; Figure 9). These results suggest that SA ameliorates midbrain dopaminergic neuronal damage and motor deficits in mice caused by LPS injection by inhibiting microglia-mediated neuro-inflammation. It also suggests the feasibility of a therapeutic strategy to mitigate inflammation-related disease processes by targeting neuro-inflammation.

## Conclusion

Overall, this experiment reveals that SA exerts neuroprotective effects in an *in vitro* and *in vivo* model of LPS injection by inhibiting neuro-inflammation. On the one hand, it suggests that SA may be a potential candidate for PD treatment, but its specific role and mechanisms need to be further explored. On the other hand, it also suggests the feasibility of finding strategies to treat PD, and indeed other inflammation-related diseases, by targeting neuro-inflammation.

## Data Availability

The original contributions presented in the study are included in the article/Supplementary materials, further inquiries can be directed to the corresponding authors.
